# Superficial heat therapy in women’s health

**DOI:** 10.3389/fmed.2026.1832193

**Published:** 2026-06-26

**Authors:** Antti Puhakka, Sylvia Mechsner, Katarzyna Placek, José Lourenço Reis, Gabriele Saccone, Angelo Cagnacci

**Affiliations:** 1 Department of Obstetrics and Gynecology, University of Helsinki and Helsinki University Hospital, Helsinki, Finland; 2 Endometriosis Centre Charité University Hospital, Berlin, Germany; 3 Clinical Department of Obstetrics, Women’s Diseases and Oncological Gynecology, Nicolaus Copernicus University, Bydgoszcz, Poland; 4 Department of Gynecology and Obstetrics, Hospital Da Luz Torres De Lisboa, Lisbon, Portugal; 5 Department of Neurosciences, Reproductive Sciences and Oral Sciences, University of Naples Federico II, Naples, Italy; 6 Head of the Department of Gynecology and Obstetrics, San Martino Hospital, University of Genoa, Genoa, Italy

**Keywords:** breastfeeding, dysmenorrhoea, endometriosis, labor pain, menopause, MSK pain, superficial heat therapy, Women’s health

## Abstract

**Background and Objective:**

Female-specific pain conditions such as dysmenorrhoea, endometriosis, and pain associated with pregnancy, childbirth, and menopause are highly prevalent and frequently impair quality of life. Superficial heat therapy (SHT) has long been used for pain relief and is well established in the management of musculoskeletal pain through mechanisms including vasodilation, improved microcirculation, muscle relaxation, and modulation of peripheral and central nociceptive pathways. This narrative review aims to synthesize current scientific evidence and clinical experience regarding the role of SHT in managing pain across multiple stages of women’s health.

**Methods:**

A targeted literature search was conducted in PubMed and Scopus for studies published between 2010 and 2026, supplemented by earlier relevant publications. Search terms included “superficial heat therapy,” “thermotherapy,” “heat wrap,” “dysmenorrhoea,” “endometriosis,” “labor pain,” “postpartum pain,” “breastfeeding-related musculoskeletal pain,” and “menopause-related musculoskeletal pain.” Evidence was evaluated and integrated with the authors’ clinical experience.

**Results:**

Evidence suggests that SHT may provide clinically meaningful pain relief in several female-specific conditions. In dysmenorrhoea and endometriosis-related pelvic pain, SHT has shown analgesic efficacy comparable in some studies to commonly used analgesics. During labor, thermal interventions such as warm compresses are associated with reduced pain perception and improved maternal comfort. In postpartum and breastfeeding contexts, SHT may alleviate lumbopelvic and musculoskeletal pain related to biomechanical stress and prolonged postures. In peri- and postmenopausal women, SHT can reduce musculoskeletal discomfort and improve functional outcomes. Wearable heat patches represent a practical option due to their sustained, controlled heat delivery and compatibility with daily activities.

**Conclusion:**

SHT represents a safe, non-invasive, and accessible adjunctive modality for the management of abdominal and MSK pain across different stages of women’s lives. Although it should not replace standard pharmacological or surgical treatments, it may complement conventional therapies and support patient self-management strategies. The integration of SHT into routine clinical care may enhance patient self-management, reduce reliance on pharmacological therapies, and improve quality of life across different stages of women’s health. Future well-designed clinical studies are needed to standardize treatment protocols, establish optimal application parameters, and further clarify its role in conditions where current evidence remains limited or largely extrapolated.

## Introduction

Pain in women is uniquely complex as it is shaped not only by biological factors, but also by sociocultural dynamics, and female-specific painful conditions such as dysmenorrhoea, endometriosis, pregnancy, childbirth, and menopause are frequently compounded by societal stigma, secrecy, myths and misinformation which contribute to a distorted and long-lasting psychological framing (1). Moreover, women’s pain is frequently normalized or minimized, both in clinical settings and in society, leading to underdiagnosis and undertreatment (2). The application of superficial heat therapy (SHT) for pain relief in women-related conditions has a long and well-documented history spanning from ancient civilizations to modern clinical practice: Egyptian papyruses describe the use of heated stones and fomentations applied to the abdomen to alleviate pelvic pain related to menstrual cramps and labor (3, 4); in 5th century BC Hippocrates of Kos recommended warm compresses and poultices for “uterine suffocation” and relieve pain associated with menstruation; in the 2nd century AD Galen advocated hot applications to relax the uterus and relieve “painful flux” (5); in the 12th century, Hildegard of Bingen recommended warm compresses infused with herbs for “women’s flux and belly pains,” while in traditional Chinese medicine (TCM), SHT including moxibustion, i.e., burning dried Artemisia near the skin, has long been used to treat dysmenorrhoea and support labor by warming meridians and dispelling “cold stagnation” (6); medical manuals and handbooks of the 1800s emphasized the application of heat to the lower abdomen to ease uterine cramping during menstruation and labor, and by the early 20th century warm baths became standard obstetric practice for labor pain relief in many hospitals (7). Moving on to more modern times, randomized controlled trials (RCTs) have demonstrated that SHT significantly reduces pain severity with an efficacy that is often comparable to that of commonly used analgesics for conditions such as dysmenorrhoea, while a growing body of evidence reports the benefits of SHT as warm water immersion and warm compresses during labor in reducing epidural analgesia use, perineal trauma and overall pain (8). From a sociological and cultural point of view, pain and any psychophysical discomfort related to typically female conditions such as labor, postpartum, menstruation, endometriosis and menopause are considered rather inevitable and acceptable, and commonly prescribed pharmacological therapies are not always effective in alleviating or resolving them, pushing women to seek remedies on their own, or to stoically endure such issues with a consequent deterioration in their quality of life (QoL) (9, 10). The goal of this paper is to evaluate the role of SHT in selected gynecological/obstetric conditions beyond menstruation by reviewing recent literature and sharing clinical practice, to foster a shared understanding among healthcare professionals as well as empower women to make informed decisions about their selfcare.

## Methods

### Evidence acquisition

This narrative review is based on a targeted literature search conducted in PubMed and Scopus for English-language studies published between 2010 and 2026, complemented by earlier articles when historically or clinically relevant, which included the following key-words: “superficial heat therapy,” “thermotherapy,” “heat wrap,” “dysmenorrhoea,” “endometriosis,” “labor pain,” “postpartum pain,” “breastfeeding-related musculoskeletal (MSK) pain,” and “menopause-related MSK pain” Randomized controlled trials, observational studies, systematic reviews, narrative reviews, and clinical guidelines were included, while conference abstracts, editorials, letters, and studies not directly relevant to the scope of the review were excluded. Titles and abstracts were screened by the authors for relevance, and full texts were assessed when necessary. The quality and strength of the available evidence were evaluated qualitatively according to study design, methodological rigor, sample size, and consistency of findings, and were classified as high, moderate, or low. This preliminary review guided the Authors’ discussion on the role of SHT in managing abdominal, pelvic, and MSK pain associated with the selected conditions, i.e., endometriosis-related pelvic and abdominal pain, premenstrual syndrome (PMS)-related dysmenorrhoea, postpartum pain, breastfeeding-related MSK pain, menopause-related MSK pain and labor pain. For each condition, the authors assessed outcomes from selected studies and integrated their clinical experience to provide clinical interpretation and practical considerations on the implementation of SHT in each specific setting, as presented in the following sections.

## Mechanism of action of superficial heat therapy

**FIGURE 1:**
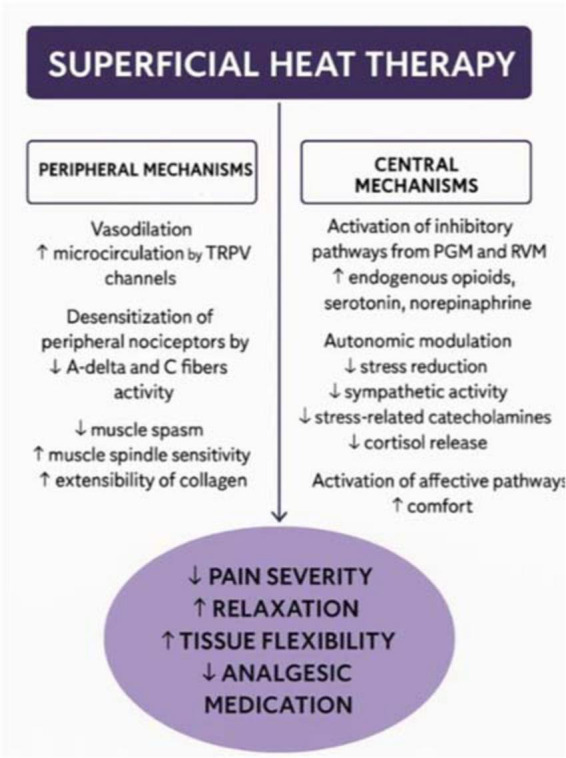
Peripheral, spinal and central mechanisms of action of SHT. TRPV, transient receptor potential vanilloid; PGM, periaqueductal grey matter; RVM, rostroventromedial medulla.

SHT exerts analgesic effects through multiple, interrelated mechanisms (Figure 1):

- Peripheral Mechanisms

✓ Activation of transient receptor potential vanilloid (TRPV) channels: local tissue heating activates thermosensitive TRPV channels promoting vasodilation and improving microcirculation. This facilitates clearance of proinflammatory mediators such as prostaglandins, bradykinin, and histamine, reducing nociceptors sensitisation. Enhanced perfusion also reduces ischemia in muscle and connective tissue, decreasing peripheral nociceptive input (11, 12);
✓ Muscle relaxation: SHT decreases muscle spindle sensitivity and increases the extensibility of collagen-rich structures, lowering muscle tone and relieving myofascial contractures, therefore leading to an improved joint and soft tissue mobility and a reduction of the pain originated in hypertonic musculature (13);
✓ Nociceptor desensitization: thermal stimulation seems to be able to transiently reduces the excitability of peripheral A-delta and C fibers, decreasing nociceptor firing rates and providing immediate analgesia (14, 15).

- Central Mechanisms

✓ Activation of descending pathways: thermal inputs activate descending analgesic systems from the periaqueductal grey matter (PGM) and rostroventromedial medulla (RVM) which release endogenous opioids, serotonin, and norepinephrine to suppress nociceptive transmission at the spinal cord level (16);
✓ Autonomic modulation: SHT reduces sympathetic activity while promoting parasympathetic dominance, attenuating the release of stress-related catecholamines and cortisol, and promoting relaxation (17);
✓ Expectations of benefit and the comforting experience of warmth enhance central pain modulation via cognitive and affective pathways, which activate cortical regions involved in descending inhibition (18).

## Modes of application of superficial heat therapy

Superficial heat therapy (SHT) can be administered through various modalities, including:

- Dry heat wraps pads or patches which are chemically or electrically activated devices (e.g., *ThermaCare^®^)* whose components reacts to air exposure delivering continuous low-level heat (∼40°C) for 8–12 h (19);
- Moist heat packs containing silica gel or clay immersed in hot water (∼70° C) wrapped in towels and applied for 15–30 min (e.g., *Hydrocollator^®^ Moist Heat Pack*s) (20);
- Wax baths containing a mixture of paraffin and mineral oil heated up to ∼50° C, applied by dipping extremities for 20–30 min *(e.g., Parabath^®^ Paraffin Heat Therapy System)* (21);
- Hot water bottles filled with water at ∼40° C and applied to affected areas for 15–30 min (22);
- Hydrotherapy by immersion of body parts in water heated to 36–40° C for 15–30 min (23);
- Infrared therapy by means of lamps emitting radiant heat at ∼50 cm distance for 15–20 min (e.g., Philips InfraCare^®^) (24).

## Use of superficial heat therapy to alleviate pain in gynecological conditions

### Endometriosis-related pelvic and abdominal pain

Endometriosis affects approximately 6–10% of women of reproductive age, and despite increased awareness recent studies reported an average delay of approximately 7 years between symptom onset and definitive diagnosis (25). The condition presents with a wide spectrum of symptoms including dysmenorrhoea, dyspareunia, dysuria, dyschezia, cyclical and acyclical pelvic pain, along with bleeding disorders and infertility, leading to emotional well-being and overall impaired QoL (26). Mechanisms underlying endometriosis-related pain involve hormonal dysregulation, inflammatory mediators, and neurovascular changes (27). Oestrogen reduction along with an increase in prostaglandin F2α (PGF2α) production sensitize peripheral nerve receptors, resulting in a lowered pain threshold and amplified pain experience (28). Elevated PGF2α levels stimulate the uterus with increased synthesis and release of oxytocin, which further promotes uterine hypercontractility and constriction of arcuate vessels, and resulting ischemia and hypoxia stimulate pain receptors in a vice circle which perpetuates neuroinflammation (29). These mechanisms contribute to a chronic pain cycle that is often resistant to conventional therapies leading to emotional distress and a meaningful impact on overall well-being (30). Currently, first-line treatment consists of hormone therapy (HT) and surgery, while pain is being treated with non-steroidal anti-inflammatory drugs (NSAIDs) and, in severe cases, opioids (31). However, more than 30% of patients are unable to achieve pain relief from conventional therapies, encouraging these treatment-resistant cohorts to look for alternative therapies (32). Several studies and reviews support the use of SHT as a non-pharmacological adjunct in managing endometriosis-related pain. A meta-analysis by *Jo J et al.* analysed six RCTs on the effect of SHT for menstrual pain highlighting its benefit when compared to acetaminophen or no intervention (33). According to the European Society of Human Reproduction and Embryology (ESHRE) given that NSAIDs and HT are the first options to be offered to patients suffering from endometriosis, non-pharmacological treatments for pain should also be discussed to improve overall wellbeing and QoL (25). As recently highlighted in a cross-sectional survey by *Werner et al.* carried out in Germany, Austria, and Switzerland, among 912 women suffering from endometriosis 75.4% reported using self-management strategies, with the most prevalent being rest (91.6%), SHT (91.1%), and exercise (63.3%) (34). Similar findings were described by *Schwartz et al.* in a retrospective evaluation of 574 women of whom 88.7% complained from endometriosis-related pain in the lower abdomen and pelvis, 62.5% reported the use of home remedies and complementary health approaches among which SHT was the most commonly used to get relief followed by rest, massage, homeopathy, phytotherapy, acupuncture and kinesiology (35).

#### Clinical interpretation and practical considerations

➢ Despite being not curative, SHT can be seen as a valuable and well-accepted adjunctive therapy to HR in endometriosis, particularly for daily symptom relief, especially in women who prefer to avoid systemic medications or who need additional options to be added to NSAIDs, and painkillers.
➢ Topical application of heat is considered safe, as it does not exacerbate underlying lesions or inflammation which are typical of the condition.
➢ Discreet, wearable heat patches (e.g., *ThermaCare^®^) are preferred over traditional methods (e.g., hot water bottles), as they allow mobility and integration into daily life as well as controlled temperature to avoid burnings.*
➢ SHT is most effective when used proactively during pain episodes in endometriosis, and it may also support emotional comfort and relaxation which play a pivotal role in the overall well-being of these complex cohorts.

### PMS-related abdominal pain

Beginning 5–10 days before menstruation, PMS features a constellation of recurrent somatic and affective symptoms that occur during the luteal phase of the menstrual cycle and resolve shortly after menstruation begins (36). PMS affects up to 20% of women of reproductive age, with 20–30% of this population experiencing moderate to severe psychological symptoms that significantly impair their daily functioning such as mood lability, anxiety, difficulty concentrating, sleep disturbances, and fatigue alongside with somatic symptoms, i.e., abdominal discomfort, breast tenderness, headache, myalgia, and a general sense of physical distress which is distinct from the cramping pain of dysmenorrhoea (37, 38). These symptoms likely result from neurohormonal fluctuations, particularly altered sensitivity to normal cycling levels of oestrogen and progesterone, along with modifications in serotonergic neurotransmission (39). Management of PMS pain focuses on symptom relief, and first-line interventions include lifestyle modifications such as regular exercise, dietary changes and stress reduction. For moderate to severe mood symptoms, selective serotonin reuptake inhibitors (SSRIs) are considered the most effective pharmacologic option, while in refractory cases and based on patient preference ovulation suppression with HT can be proposed (40). SHT can be considered as a treatment option for abdominal discomfort and related MSK pain associated with PMS due to its mechanisms of action, i.e., local vasodilation, increased tissue blood flow, and muscle relaxation, which have primarily been assessed in dysmenorrhoea. RCTs and systematic reviews in dysmenorrhoea populations have demonstrated that SHT reduces pain intensity and improves pressure pain thresholds in the abdominal region, with comparable efficacy to NSAIDs and superior to acetaminophen for short-term relief (41). In a recent systematic review *Yuan et al.* found that HT was likely to reduce pain intensity both during prophylaxis and acute episodes in primary dysmenorrhea and with comparable analgesic efficacy and a superior safety profile compared to NSAIDs, while *Akin et al.* previously demonstrated that continuous, low-level topical heat wrap therapy was superior to acetaminophen for pain relief in dysmenorrhoea while reducing fatigue and mood swings (42, 43). *Machado et al.* showed that thermotherapy reduces pain intensity and increases abdominal pressure pain threshold in dysmenorrhoea, suggesting a relevant mechanism which may be relevant to PMS-related abdominal and MSK discomfort (44).

#### Clinical interpretation and practical considerations

➢ PMS is a complex syndrome which impairs daily functioning, with a broad spectrum of physical manifestations from bloating-related abdominal discomfort, to mastalgia and MSK aches, along with distressing psychological issues. The control over the physical symptoms of this condition certainly has undeniable positive repercussions on the psychological aspects that are associated with it.
➢ The Panel stresses that there are currently no specific published data on the use of SHT in PMS-related abdominal and MSK pain. However, data extrapolation from available research on dysmenorrhoea is reasonable based on the known mechanism of action of SHT. Indeed, studies mainly focus on the use of SHT in menstrual pain, and the underlying principle may indirectly justify its use for PMS-related somatic pain.
➢ Therefore, it could be stated that SHT is a reasonable, low-risk, nonpharmacologic option for abdominal and MSK pain associated with PMS, supported by indirect evidence from the available literature on MSK pain and dysmenorrhoea in other populations, and the Panel sees no reason for not using SHT in this clinical context as well, due to its favorable safety profile.
➢ On the other hand, the absence of any specific data on this topic encourages the opening of a new and intriguing direction for research to assess the use of SHT also in other unexplored clinical contexts, and dedicated clinical studies are warranted to directly evaluate the efficacy and optimal use of this therapeutic methodology in women with PMS-related abdominal and MSK symptoms.

### Labor pain

Labor pain is a multidimensional experience resulting from the integration of physiological, neuroanatomical, and psychological factors. During the first stage of labor, pain originates primarily from uterine contractions and cervical dilatation, which activate both mechano- and chemo-sensitive nociceptors in the uterus and cervix whose signals are transmitted via visceral afferents accompanying sympathetic nerves, entering the spinal cord at T10-L1 levels (45). Myometrial ischemia during contractions leads to the release of neurochemical mediators such as bradykinin, prostaglandins, and substance P, which sensitize nociceptors and amplify pain perception (46). As labor progresses to the second stage, pain shifts to a somatic origin due to distension and compression of the vagina, perineum, and pelvic floor, and painful signals are transmitted through the pudendal nerve, entering the spinal cord at S2-S4 levels (47, 48). Psychological factors such as anxiety, fear, and previous labors further modulate pain perception through descending pathways and endogenous opioid systems whose effectiveness varies among individuals, while proinflammatory cytokines and transient hypoxia during labor can enhance nociceptor sensitivity and contribute to pain intensity (49). The integration of physical, psychological, and neurochemical factors underscores the need for a multimodal approach to labor pain management (50). SHT emerges from available literature as a safe adjunct for labor pain relief, particularly in the first stage, and may help attenuate the transmission and perception of nociceptive signals, promoting local vasodilation, reducing muscle tension, and possibly modulating spinal and supraspinal pain pathways. In the 1980s *Khamis et al.* observed that when a hot water bag was applied on the abdominal wall of 15 full-term multiparous women for 20 min in the first stage of labor and removed for another 20 min, SHT resulted in a significant increase in uterine activity measured by cardiotocography, concluding that SHT had the potential to safely stimulate labor while aiding in reaching an overall maternal comfort and pain control (51). In more recent times the role of SHT in labor and birth outcomes have thoroughly been investigated. *Smith et al*. carried out a Cochrane review and meta-analysis to examine the evidence on unconventional methods for labor pain management including 14 RCTs for a total of about 2000 women and concluded that massage, SHT and thermal manual methods are effective in reducing pain, length of labor while improving women’s sense of control and overall emotional experience (52). These findings were confirmed by another systematic review and meta-analysis by *Goswami et al*. who examined 10 RCTs on the effect of SHT on pain intensity, and duration of labor, concluding that SHT was significantly effective in reducing pain intensity in the first stage of labor, decreasing its duration and newborns showed better Apgar scores at the 5th minute compared to standard therapies (53). These findings have been backed up by a number of experimental studies such as the research by *Kaur et al*. who evaluated the effectiveness of SHT by means of a hydrocollator pack applied on the lumbo-sacral region of 88 nulliparous parturients for 20 min for 3 times with 1 h interval starting from 4 to 5 cm of cervical dilatation and found that labor pain intensity scores were significantly lower in the experimental group than among controls, and no significant difference were recorded as to any fetal or infant-related safety findings (54). *Gaheen et al.* evaluated the effect of perineal massage, warm compresses and hands on techniques during the second stage of labor on perineal outcomes by randomizing 120 parturients, and pointed out that perineal pain intensity as well as perineal tear were significantly reduced among women who received the described treatment compared to control group (55). Similar findings were described by *Dahlen et al.* who showed that warm compresses applied on the perineum for no longer than 30 min with a 30 to 60 min break significantly reduced third- and fourth-degree tears (56).

#### Clinical interpretation and practical considerations

➢ SHT has been successfully used during labor to help women managing pain while increasing comfort, especially during the first stage, emerging as a long-established method that can improve the overall experience.
➢ SHT positively stimulates uterine muscle activity, potentially reducing labor time, facilitating relaxation, optimizing perineal outcomes and alleviating postpartum issues.
➢ The use of SHT is generally considered safe for the fetus, although superficial heating devices should allow for constant monitoring of the temperature applied.
➢ SHT is not recommended during epidural anesthesia, and should not interfere with any obstetric procedures or emergency intervention that may be necessary during labor.

### Post-partum pain

Postpartum pain is a frequent, multifactorial condition arising from childbirth-related biomechanical stress, hormonal shifts, neuromuscular dysfunction, and tissue injury (57). Although acute perineal and uterine pain typically resolve within weeks, persistent lumbopelvic pain (PLP) localized between the iliac crest, gluteal folds, sometimes radiating to the posterior thigh, and pelvic girdle pain (PGP) particularly near the sacroiliac joints sometimes radiating to the posterior thigh, can impair maternal function and QoL for months to years postpartum (57). Several mechanisms underlie postpartum MSK pain and nociceptive syndromes, since high relaxin and progesterone levels induce ligamentous laxity in the sacroiliac and pelvic ligaments, while neuromuscular adaptations and tissue injury contribute to pain persistence. Uterine cramping pain is a common postpartum complaint, resulting from involutional contractions of the uterus. Building on the multimodal framework for other women’s health condition, the most robust evidence for acute uterine cramping pain after vaginal delivery supports the use of NSAIDs as first-line pharmacologic therapy, while acetaminophen may be used as an adjunct though its efficacy is less certain (58). For acute perineal pain after vaginal delivery with trauma, cold therapy (i.e., ice packs) is recommended as first-line treatment, while a short course of low-dose epidural morphine may be considered with appropriate monitoring for women with severe perineal trauma (59). Nonpharmacologic adjuncts such as SHT in the form of heating pads applied to the lower abdomen are recommended by the American College of Obstetricians and Gynecologists (ACOG) as part of a stepwise approach, particularly for after-birth pain, based on consensus and clinical experience, although the supporting evidence is of low certainty and primarily extrapolated from dysmenorrhoea literature (58).

#### Clinical interpretation and practical considerations

➢ For women with persistent lumbopelvic pain localized between the iliac crest and gluteal folds, or pelvic girdle pain near the sacroiliac joints arising post-partum, literature supports a multimodal strategy emphasizing nonpharmacologic interventions.
➢ The available evidence supporting the use of SHT in postpartum pain remains limited and is largely extrapolated from studies conducted in dysmenorrhoea and non-specific MSK pain populations. Further well-designed clinical trials are needed to establish its effectiveness, safety, and optimal application protocols in postpartum women. However, SHT may be considered a low-risk adjunct for symptom relief, particularly in combination with other modalities, especially to minimize medication exposure or as part of a patient-centered decision-making approach. SHT should not be used in case of caesarean delivery or during epidural analgesia, and caution should be exercised when using SHT in the first 24–48 h after delivery due to the potential risks of postpartum bleeding and uterine atony. Although SHT does not directly affect coagulation, the immediate postpartum period is characterized by a heightened risk of primary postpartum hemorrhage most commonly due to uterine atony, and the uterus remains highly vascular during this time.

### Breastfeeding-related MSK pain

Breastfeeding-related MSK pain is highly prevalent, and recent survey data indicate that up to 84% of breastfeeding mothers experience back pain at least once a month, while nearly half report weekly pain in the cervical, thoracic, or lumbosacral regions (60). Dysfunctional states, including moderate disability, are present in over a quarter of those affected, as prolonged breastfeeding sessions with suboptimal positioning, particularly sitting without adequate support, are associated with increased pain intensity, while lying or semi-lying positions with head support are associated with less neck pain (60). Breastfeeding-related pain, including MSK discomfort, is a leading cause of early weaning, reduced breastfeeding duration, and it interferes with activities of daily living while negatively affecting QoL (61). RCTs and meta-analyses demonstrate that SHT reduces pain and disability in non-specific acute and subacute low back pain (62, 63). Heat wrap therapy applied for several hours daily over 5–7 days has shown greater pain reduction compared to oral placebo and immediate pain relief, and the addition of exercise to SHT further improved pain and function (64). *Nadler et al.* conducted two RCTs on continuous low-level SHT in individuals with acute non-specific lower back pain and reported significantly greater pain relief than commonly used analgesics (i.e., acetaminophen and ibuprofen) and placebo for up to 16 h, along with improved mobility and reduced muscle stiffness following treatment (65). Moreover, additional beneficial outcomes on SHT applied also to other MSK regions, such as the quadriceps, the plantar fascia and the knee, were reported by Petrofsky et al. (66–69).

#### Clinical interpretation and practical considerations

➢ Breastfeeding women frequently complain of MSK pain due to prolonged and unergonomic positions. In addition, MSK pain in this context can be exacerbated by emotional and psychological issues, making new mothers particularly sensitive and vulnerable.
➢ A multimodal approach integrating ergonomic support, targeted exercise, safe pharmacologic analgesia, education, and adjunctive SHT can improve maternal comfort, functional capacity, and breastfeeding duration in lactating women during the first 6 months postpartum.
➢ Evidence highlighting SHT efficacy in successfully treating non-specific MSK pain arising in other clinical contexts and affecting other body parts might also prove relevant and provide a solid base for implementing this treatment modality in breastfeeding-related MSK discomfort, where static postures and muscle fatigue are common, and patients may prefer to avoid systemic medications.
➢ Although there are no contraindications to the application of SHT to the painful area, it is important to avoid contact with the skin of the breast, which is normally tense and sensitive during breastfeeding.

### Menopause-related MSK pain

More than 70% of women undergoing the menopause transition will experience MSK symptoms and 25% will develop some degree of disability because of them. Often-unrecognized, menopause-related MSK symptoms involve the neck, shoulders, low back, and the large joints (e.g., knees, hips), contributing to reduced mobility, sleep disturbances, diminished QoL, and women experience a linear increase in moderate to severe MSK pain from pre-menopause through perimenopause to post-menopause (70, 71). These symptoms, including arthralgia, loss of muscle mass and bone density, and progression of osteoarthritis are largely driven by oestrogen fluctuations and deficiency which increase inflammation and heighten pain sensitivity, and they may be misattributed to aging or other comorbidities delaying correct diagnosis (72, 73). Management strategies are multidisciplinary and lifestyle interventions are pivotal since regular weight-bearing and resistance exercise are associated with preservation of muscle and bone mass and overall reduction in symptom severity (74). Nutritional optimization (i.e., adequate protein, calcium, and vitamin D intake) is essential for bone and muscle health, and conservative measures such as simple analgesia, weight loss, and physical therapy should be encouraged (75). HT can be considered for MSK symptoms especially in women < 60 years of age or within 10 years of menopause onset with vasomotor manifestations, while for those with osteoporosis but without any vasomotor symptoms, non-hormonal osteoporosis medications are preferred (72–74). SHT is a safe, low-risk adjunct that provides relief of MSK pain in peri- and postmenopausal women, even though it does not directly address loss of muscle mass or bone density, nor does it modify disease progression. However, SHT (e.g., hot packs, heat wraps, hydrotherapy) is reported to be able to reduce pain intensity and improve function in rheumatic and MSK pain which can accompany several conditions, including menopausal arthralgia and osteoarthritis, with a favorable safety profile (76–79).

#### Clinical interpretation and practical considerations

➢ The clinical picture of menopause is extremely varied, therefore the occurrence of MSK pain in this context often makes part of a wider and more complex syndrome.
➢ Since its mechanism of action involves local vasodilation, reduced muscle spasm, and modulation of inflammatory mediators, SHT may be used as part of a multimodal approach for symptomatic pain relief, especially in women with osteoarthritis or chronic joint pain during menopause.
➢ Addressing comorbidities which are a feature of the menopause transition such as sleep disturbance, depression, and fatigue, is also important for improving QoL in these cohorts of patients. Furthermore, patients’ education about the MSK syndrome of menopause along with a proactive management is critical to prevent long-term disability.
➢ In individuals suffering from hot flushes, the application of SHT may exacerbate or worsen these symptoms. Therefore, SHT should be evaluated on a case-by-case basis especially in patients with vasomotor manifestations.

## Safety considerations

To minimize the risk of adverse effects, heat should generally be applied within the range of 38–42 °C, and prolonged continuous use should not exceed 8–12 h when wearable low-level heat devices are used. The main potential adverse effects of SHT include skin irritation, erythema, overheating, discomfort, and burns, particularly when heat is applied for prolonged periods, at excessive temperatures, or over areas with impaired sensation. Therefore, SHT should not be applied over open wounds, inflamed or damaged skin, infected areas, or regions with reduced thermal sensitivity. Patients should be advised to discontinue treatment in case of pain, burning sensation, marked redness, dizziness, or worsening symptoms. In endometriosis-related and PMS-related abdominal pain, SHT may be used as an adjunctive self-management strategy, provided that heat is not applied over damaged or irritated skin. In labor, warm compresses or local heat application may improve maternal comfort, but temperature should be carefully monitored and the intervention should not interfere with obstetric monitoring or emergency procedures. SHT is not recommended during epidural or spinal analgesia because reduced sensation may increase the risk of thermal injury. In the postpartum period, SHT should be used cautiously, particularly during the first 24–48 h after delivery, due to the physiological risk of postpartum bleeding and uterine atony. It should not be applied over caesarean wounds, episiotomy sites, perineal trauma, or any area of damaged tissue. In breastfeeding-related musculoskeletal pain, heat may be applied to the cervical, thoracic, or lumbar regions, but direct application to the breast should be avoided, especially during active breastfeeding, and infant skin contact with heated devices should be prevented. Finally, in peri- and postmenopausal women, SHT should be considered on a case-by-case basis in women with vasomotor symptoms, as heat exposure may exacerbate hot flushes.

## Conclusion

Superficial heat therapy (SHT) emerges as a clinically relevant, non-invasive, and accessible modality with a robust documented efficacy to manage MSK and abdominal pain across a spectrum of gynecological and obstetric conditions (Table 1). SHT modulates pain through peripheral, spinal, and central mechanisms, offering a multidimensional approach in clinical settings such as dysmenorrhoea, endometriosis, PMS-related pain, labor and postpartum pain, breastfeeding and menopause-related MSK pain. The integration of SHT into routine clinical care represents a patient-centered approach that is especially valuable given the limitations and side effects of long-term pharmacologic therapies. Heating modalities such as wearable patches provide prolonged and discreet relief, aligning well with the daily demands of women in various life stages. Importantly, SHT also supports self-management strategies and empowers women to take active roles in their health, potentially improving their QoL and reducing healthcare burden. Future research should aim to standardize SHT protocols across indications, assess long-term outcomes, patient adherence, cost-effectiveness in diverse healthcare settings, and explore new clinical settings where this treatment modality might be applicable.

**TABLE 1:** Clinical use of SHT in female-related conditions.

Condition	Expected benefits	Recommended temperature and application	Duration/ frequency	Contraindications/ warnings	Evidence level	Clinical notes
Endometriosis	Relief of pelvic and abdominal pain; muscle relaxation	38–40 °C	8–12 h continuous use	Avoid on inflamed or damaged skin	Moderate	Adjunctive use only; compatible with daily activities
PMS-related pain	Reduction of cramps and MSK discomfort	38–42 °C, lower abdomen or lumbar area	15–30 min or intermittent daily use	Not effective for psychological symptoms	Low–Moderate (indirect evidence)	Evidence extrapolated from dysmenorrhoea studies
Labour pain	Pain relief during first stage; improved comfort	Warm compresses ~40 °C, abdomen, back, perineum	20–30 min with intervals	Avoid during epidural/spinal analgesia	Moderate	May improve labour outcomes and maternal comfort
Postpartum pain	Relief of uterine cramps and lumbopelvic discomfort	38–40 °C, abdominal or back application	After first 24–48 h postpartum	Avoid on surgical wounds or damaged tissue	Low (indirect evidence)	Use cautiously due to bleeding/uterine atony risk
Breastfeeding-related MSK pain	Relief of neck, shoulder, and back pain	38–42 °C, cervical, thoracic, lumbar areas	15–30 min or prolonged low-level application	Avoid breast area and infant skin contact	Moderate (extrapolated MSK evidence)	Combine with ergonomic education and physiotherapy
Menopause-related MSK pain	Reduction of joint stiffness and pain	38–42 °C, major joints, back, knees	15–30 min daily or as needed	May worsen hot flushes	Low–Moderate	Best used within multimodal management

hrs, hours; MSK, musculoskeletal; PMS, pre-menstrual syndrome; NSAIDs, nonsteroidal anti-inflammatory drugs.

## Study limitations

Patients’ responses to SHT are variable and influenced by psychological, hormonal, and cultural factors. Moreover, literature search has highlighted that there is a lack of standardized protocols regarding temperature, duration, and frequency of application of this treatment modality, while high-quality RCTs are limited for many gynecological applications of SHT.

## References

[1] Roos M Wimmelbacher V Klein L Kesiæ M Rueß AK Necker C et al. Real-world evidence shows gaps in awareness, medical help-seeking, and diagnosis for primary dysmenorrhea but not premenstrual syndrome: cross-sectional observational study. *J Med Internet Res* (2025) 27:e68148. 10.2196/68148 40934486 PMC12425425

[2] Eitze S Reinhardt A. Keep period pain a secret? Expanding the theory of planned behavior with endometriosis knowledge and menstrual stigma to explain women’s intentions to talk about menstrual discomfort. *Health Psychol.* (2025) 44:1028–38. 10.1037/hea0001502 40272413

[3] Smith L. The Kahun gynaecological papyrus: ancient Egyptian medicine. *J Fam Plann Reprod Health Care.* (2011) 37:54–5. 10.1136/jfprhc.2010.0019 21367707

[4] Patabendige M Rolnik DL Li W Weeks AD Mol BW. How labor induction methods have evolved throughout history, from the Egyptian era to the present day: evolution, effectiveness, and safety. *Am J Obstet Gynecol MFM*. (2025) 7:101515. 10.1016/j.ajogmf.2024.101515 39447696

[5] Totelin L. Old recipes, new practice? The Latin adaptations of the hippocratic gynaecological treatises. *Soc Hist Med.* (2011) 24:74–91. 10.1093/shm/hkq103

[6] Xu J Deng H Shen X. Safety of moxibustion: a systematic review of case reports. *Evid Based Complement Alternat Med*. (2014) 2014:783704. 10.1155/2014/783704 24976851 PMC4058265

[7] Purcal NK. The politics of midwifery education and training in New South Wales during the last decades of the 19th Century. *Women Birth.* (2008) 21:21–5. 10.1016/j.wombi.2007.11.002 18155976

[8] Freiwald J Magni A Fanlo-Mazas P Paulino E Sequeira de Medeiros L Moretti B et al. A role for superficial heat therapy in the management of non-specific, mild-to-moderate low back pain in current clinical practice: a narrative review. *Life (Basel).* (2021) 11:780. 10.3390/life11080780 34440524 PMC8401625

[9] Maghalian M Alikamali M Nabighadim M Mirghafourvand M. The effects of warm perineal compress on perineal trauma and postpartum pain: a systematic review with meta-analysis and trial sequential analysis. *Arch Gynecol Obstet.* (2024) 309:843–69. 10.1007/s00404-023-07195-2 37632600

[10] Lamvu G Carrillo J Ouyang C Rapkin A. Chronic pelvic pain in women: a review. *JAMA.* (2021) 325:2381–91. 10.1001/jama.2021.2631 34128995

[11] Brunt VE Eymann TM Francisco MA Howard MJ Minson CT. Passive heat therapy improves cutaneous microvascular function in sedentary humans via improved nitric oxide-dependent dilation. *J Appl Physiol* (1985) 2016:716–23. 10.1152/japplphysiol.00424.2016 27418688 PMC6195670

[12] Li WW Zhao Y Liu HC Liu J Chan SO Zhong YF et al. Roles of thermosensitive transient receptor channels TRPV1 and TRPM8 in paclitaxel-induced peripheral neuropathic pain. *Int J Mol Sci*. (2024) 25:5813. 10.3390/ijms25115813 38892000 PMC11171746

[13] Sneddon LU. Comparative physiology of nociception and pain. *Physiology (Bethesda).* (2018) 33:63–73. 10.1152/physiol.00022.2017 29212893

[14] Lin M Genin GM Xu F Lu T. Thermal pain in teeth: electrophysiology governed by thermomechanics. *Appl Mech Rev.* (2014) 66:308011–3080114. 10.1115/1.4026912 25516631 PMC4240033

[15] Ventriglia G Gervasoni F Franco M Magni A Panico G Iolascon G. Musculoskeletal pain management and thermotherapy: an exploratory analysis of Italian physicians’ attitude, beliefs, and prescribing habits. *J Pain Res.* (2023) 16:1547–57. 10.2147/JPR.S401550 37197390 PMC10184851

[16] Giniatullin R. Ion channels of nociception. *Int J Mol Sci.* (2020) 21:3553. 10.3390/ijms21103553 32443485 PMC7278920

[17] Lumley MA Cohen JL Borszcz GS Cano A Radcliffe AM Porter LS et al. Pain and emotion: a biopsychosocial review of recent research. *J Clin Psychol.* (2011) 67:942–68. 10.1002/jclp.20816 21647882 PMC3152687

[18] Malanga GA Yan N Stark J. Mechanisms and efficacy of heat and cold therapies for musculoskeletal injury. *Postgrad Med.* (2015) 127:57–65. 10.1080/00325481.2015.992719 25526231

[19] Petrofsky JS Laymon M Berk L Bains G. Effect of thermacare heatwraps and icy hot cream/patches on skin and quadriceps muscle temperature and blood flow. *J Chiropr Med.* (2016) 15:9–18. 10.1016/j.jcm.2015.12.002 27069427 PMC4812039

[20] Lohman EB III Bains GS Lohman T DeLeon M Petrofsky JS. A comparison of the effect of a variety of thermal and vibratory modalities on skin temperature and blood flow in healthy volunteers. *Med Sci Monit.* (2011) 17:MT72–81. 10.12659/msm.881921 21873956 PMC3560507

[21] Dilek B Gözüm M Şahin E Baydar M Ergör G El O et al. Efficacy of paraffin bath therapy in hand osteoarthritis: a single-blinded randomized controlled trial. *Arch Phys Med Rehabil.* (2013) 94:642–9. 10.1016/j.apmr.2012.11.024 23187044

[22] Uchiyama-Tanaka Y. Effect of thermal therapy using hot water bottles on brain natriuretic peptide in chronic hemodialysis patients. *Cardiol Ther.* (2012) 1:2. 10.1007/s40119-012-0002-z 25135156 PMC4107443

[23] Jackman JS Bell PG Van Someren K Gondek MB Hills FA Wilson LJ et al. Effect of hot water immersion on acute physiological responses following resistance exercise. *Front Physiol*. (2023) 14:1213733. 10.3389/fphys.2023.1213733 37476688 PMC10354234

[24] Tsagkaris C Papazoglou AS Eleftheriades A Tsakopoulos S Alexiou A Gãman MA et al. Infrared radiation in the management of musculoskeletal conditions and chronic pain: a systematic review. *Eur J Investig Health Psychol Educ*. (2022) 12:334–43. 10.3390/ejihpe12030024 35323210 PMC8946909

[25] Becker CM Bokor A Heikinheimo O Horne A Jansen F Kiesel L et al. ESHRE guideline: endometriosis. *Hum Reprod Open*. (2022) 2022:hoac009. 10.1093/hropen/hoac009 35350465 PMC8951218

[26] Culley L Law C Hudson N Denny E Mitchell H Baumgarten M et al. The social and psychological impact of endometriosis on women’s lives: a critical narrative review. *Hum Reprod Update*. (2013) 19:625–39. 10.1093/humupd/dmt027 23884896

[27] Wang PH Yang ST Chang WH Liu CH Lee FK Lee WL. Endometriosis: part I. Basic concept. *Taiwan J Obstet Gynecol*. (2022) 61:927–34. 10.1016/j.tjog.2022.08.002 36427994

[28] Carlyle D Khader T Lam D Vadivelu N Shiwlochan D Yonghee C. Endometriosis pain management: a review. *Curr Pain Headache Rep*. (2020) 24:49. 10.1007/s11916-020-00884-6 32671581

[29] Maddern J Grundy L Castro J Brierley SM. Pain in endometriosis. *Front Cell Neurosci*. (2020) 14:590823. 10.3389/fncel.2020.590823 33132854 PMC7573391

[30] Gstoettner M Wenzl R Radler I Jaeger M. I think to myself ‘why now?”’ – a qualitative study about endometriosis and pain in Austria. *BMC Womens Health*. (2023) 23:409. Erratum in: BMC Womens Health. 2023;23:455. doi: 10.1186/s12905-023-02610-x 10.1186/s12905-023-02576-w 37542309 PMC10403941

[31] Brichant G Laraki I Henry L Munaut C Nisolle M. New therapeutics in endometriosis: a review of hormonal, non-hormonal, and non-coding RNA treatments. *Int J Mol Sci.* (2021) 22:10498. 10.3390/ijms221910498 34638843 PMC8508913

[32] Chen C Li X Lu S Yang J Liu Y. Acupuncture for clinical improvement of endometriosis-related pain: a systematic review and meta-analysis. *Arch Gynecol Obstet*. (2024) 310:2101–14. 10.1007/s00404-024-07675-z 39110208 PMC11393010

[33] Jo J Lee SH. Heat therapy for primary dysmenorrhea: a systematic review and meta-analysis of its effects on pain relief and quality of life. *Sci Rep.* (2018) 8:16252. 10.1038/s41598-018-34303-z 30389956 PMC6214933

[34] Werner F Jasinski V Voltolini Velho R Sehouli J Mechsner S. The role of self-management in endometriosis pain: insights from a cross-sectional survey in Germany, Austria, and Switzerland. *Arch Gynecol Obstet.* (2025) 312:425–34. 10.1007/s00404-025-08019-1 40253561 PMC12334507

[35] Schwartz ASK Gross E Geraedts K Rauchfuss M Wölfler MM Häberlin F et al. The use of home remedies and complementary health approaches in endometriosis. *Reprod Biomed Online*. (2019) 38:260–71. 10.1016/j.rbmo.2018.10.009 30612955

[36] Yonkers KA O’Brien PM Eriksson E. Premenstrual syndrome. *Lancet*. (2008) 371:1200–10. 10.1016/S0140-6736(08)60527-9 18395582 PMC3118460

[37] Dilbaz B Aksan A. Premenstrual syndrome, a common but underrated entity: review of the clinical literature. *J Turk Ger Gynecol Assoc*. (2021) 22:139–48. 10.4274/jtgga.galenos.2021.2020.0133 33663193 PMC8187976

[38] Tiranini L Nappi RE. Recent advances in understanding/management of premenstrual dysphoric disorder/premenstrual syndrome. *Fac Rev*. (2022) 11:11. 10.12703/r/11-11 35574174 PMC9066446

[39] Barcikowska Z Rajkowska-Labon E Grzybowska ME Hansdorfer-Korzon R Zorena K. Inflammatory markers in dysmenorrhea and therapeutic options. *Int J Environ Res Public Health*. (2020) 17:1191. 10.3390/ijerph17041191 32069859 PMC7068519

[40] Shulman LP. Gynecological management of premenstrual symptoms. *Curr Pain Headache Rep*. (2010) 14:367–75. 10.1007/s11916-010-0131-9 20665250

[41] Kannan P Claydon LS. Some physiotherapy treatments may relieve menstrual pain in women with primary dysmenorrhea: a systematic review. *J Physiother*. (2014) 60:13–21. 10.1016/j.jphys.2013.12.003 24856936

[42] Yuan D Liu Y Chen Z Hu Z Li X Zhang W et al. Heat therapy for primary dysmenorrhea: a systematic review and meta-analysis. *Front Med (Lausanne).* (2026) 12:1730505. 10.3389/fmed.2025.1730505 41657584 PMC12876241

[43] Akin M Price W Rodriguez G Jr. Erasala G Hurley G Smith RP. Continuous, low-level, topical heat wrap therapy as compared to acetaminophen for primary dysmenorrhea. *J Reprod Med*. (2004) 49:739–45. 15493566

[44] Machado AFP Perracini MR Rampazo ÉP Driusso P Liebano RE. Effects of thermotherapy and transcutaneous electrical nerve stimulation on patients with primary dysmenorrhea: a randomized, placebo-controlled, double-blind clinical trial. *Complement Ther Med.* (2019) 47:102188. 10.1016/j.ctim.2019.08.022 31779988

[45] Shnol H Paul N Belfer I. Labor pain mechanisms. *Int Anesthesiol Clin*. (2014) 52:1–17. 10.1097/AIA.0000000000000019 24946040

[46] Eltzschig HK Lieberman ES Camann WR. Regional anesthesia and analgesia for labor and delivery. *N Engl J Med*. (2003) 348:319–32. 10.1056/NEJMra021276 12540646

[47] Lowe NK. The nature of labor pain. *Am J Obstet Gynecol*. (2002) 186:S16–24. 10.1067/mob.2002.121427 12011870

[48] Society for Maternal-Fetal Medicine (SMFM). Electronic address: pubs@smfm.org, Society of Family Planning (SFP), Norton ME Cassidy A Ralston SJ Chatterjee D et al. Society for Maternal-Fetal Medicine Consult Series #59: The use of analgesia and anesthesia for maternal-fetal procedures. *Am J Obstet Gynecol.* (2021) 225:B2–8. 10.1016/j.ajog.2021.08.031 34461076

[49] Labor S Maguire S. The pain of labour. *Rev Pain.* (2008) 2:15–9. 10.1177/204946370800200205 26526404 PMC4589939

[50] Nori W Kassim MAK Helmi ZR Pantazi AC Brezeanu D Brezeanu AM et al. Non-pharmacological pain management in labor: a systematic review. *J Clin Med*. (2023) 12:7203. 10.3390/jcm12237203 38068274 PMC10707619

[51] Khamis Y Shaala S Damarawy H Romia A Toppozada M. Effect of heat on uterine contractions during normal labor. *Int J Gynaecol Obstet*. (1983) 21:491–3. 10.1016/0020-7292(83)90041-3 6141112

[52] Smith CA Levett KM Collins CT Dahlen HG Ee CC Suganuma M. Massage, reflexology and other manual methods for pain management in labour. *Cochrane Database Syst Rev.* (2018) 3:CD009290. 10.1002/14651858.CD009290.pub3 29589380 PMC6494169

[53] Goswami S Jelly P Sharma SK Negi R Sharma R. The effect of heat therapy on pain intensity, duration of labor during first stage among primiparous women and Apgar scores: a systematic review and meta-analysis. *Eur J Midwifery*. (2022) 6:66. 10.18332/ejm/156487 36474673 PMC9703937

[54] Kaur J Sheoran P Kaur S Sarin J. Effectiveness of warm compression on Lumbo-Sacral region in terms of labour pain intensity and labour outcomes among nulliparous: an interventional study. *J Caring Sci*. (2020) 9:9–12. 10.34172/jcs.2020.002 32296653 PMC7146727

[55] Gaheen MA Abo-Hatab T. Effect of utilizing perineal massage, warm compresses and hands on techniques during the second stage of labor on perineal outcomes. *Tanta Sci Nurs J.* (2021) 23:36–60. 10.21608/tsnj.2021.210228

[56] Dahlen HG Homer CS Cooke M Upton AM Nunn R Brodrick B. Perineal outcomes and maternal comfort related to the application of perineal warm packs in the second stage of labor: a randomized controlled trial. *Birth*. (2007) 34:282–90. 10.1111/j.1523-536X.2007.00186.x 18021143

[57] Valinger Aggeryd K Bergström C Mogren I Persson M. A limited life - a mixed methods study on living with persistent pregnancy-related lumbopelvic pain more than 12 years postpartum in Sweden. *Disabil Rehabil.* (2022) 44:3062–70. 10.1080/09638288.2020.1852447 33511884

[58] ACOG. ACOG committee opinion no. 742 summary: postpartum pain management. *Obstet Gynecol*. (2018) 132:252–3. 10.1097/AOG.0000000000002711 29939935

[59] Luxey X Lemoine A Dewinter G Joshi GP Le Ray C Raeder J et al. Acute pain management after vaginal delivery with perineal tears or episiotomy. *Reg Anesth Pain Med.* (2025) 50:503–13. 10.1136/rapm-2024-105478 38772634

[60] Ratajczak M Górnowicz R. The influence of breastfeeding factors on the prevalence of back and neck pain: data from an online survey. *BMC Musculoskeletal Disorders*. (2024) 25:675. 10.1186/s12891-024-07785-4 39210354 PMC11360292

[61] ACOG. Pharmacologic stepwise multimodal approach for postpartum pain management: ACOG clinical consensus no. 1. *Obstet Gynecol*. (2021) 138:507–17. 10.1097/AOG.0000000000004517 34412076

[62] Hegmann KT Travis R Andersson GBJ Belcourt RM Carragee EJ Donelson R et al. Non-invasive and minimally invasive management of low back disorders. *J Occup Environ Med.* (2020) 62:e111–38. 10.1097/JOM.0000000000001812 31977923

[63] Rizzo RR Cashin AG Wand BM Ferraro MC Sharma S Lee H et al. Non-pharmacological and non-surgical treatments for low back pain in adults: an overview of Cochrane reviews. *Cochrane Database Syst Rev.* (2025) 3:CD014691. 10.1002/14651858.CD014691.pub2 40139265 PMC11945228

[64] French SD Cameron M Walker BF Reggars JW Esterman AJ. A cochrane review of superficial heat or cold for low back pain. *Spine.* (2006) 31:998–1006. 10.1097/01.brs.0000214881.10814.64 16641776

[65] Nadler SF Steiner DJ Erasala GN Hengehold DA Hinkle RT Beth Goodale M et al. Continuous low-level heat wrap therapy provides more efficacy than ibuprofen and acetaminophen for acute low back pain. *Spine (Phila Pa 1976)* (2002) 27:1012–7. 10.1097/00007632-200205150-00003 12004166

[66] Nadler SF Steiner DJ Petty SR Erasala GN Hengehold DA Weingand KW. Overnight use of continuous low-level heatwrap therapy for relief of low back pain. *Arch Phys Med Rehabil.* (2003) 84:335–42. 10.1053/apmr.2003.50103 12638100

[67] Petrofsky J Laymon M Lee H. Local heating of trigger points reduces neck and plantar fascia pain. *J Back Musculoskelet Rehabil*. (2020) 33:21–8. 10.3233/BMR-181222 31594202

[68] Petrofsky J Berk L Bains G Khowailed IA Hui T Granado M et al. Moist heat or dry heat for delayed onset muscle soreness. *J Clin Med Res*. (2013) 5:416–25. 10.4021/jocmr1521w 24171053 PMC3808259

[69] Petrofsky JS Laymon MS Alshammari FS Lee H. Use of low level of continuous heat as an adjunct to physical therapy improves knee pain recovery and the compliance for home exercise in patients with chronic knee pain: a randomized controlled trial. *J Strength Cond Res*. (2016) 30:3107–15. 10.1519/JSC.0000000000001409 27776079

[70] Sipilä S Törmäkangas T Sillanpää E Aukee P Kujala UM Kovanen V et al. Muscle and bone mass in middle-aged women: role of menopausal status and physical activity. *J Cachexia Sarcopenia Muscle*. (2020) 11:698–709. 10.1002/jcsm.12547 32017473 PMC7296268

[71] Wang X Yang D Li J Jin L Xia S Jin F. Association between menopause-related symptoms and muscle mass index among perimenopausal and postmenopausal women and the mediating role of estrogen levels. *Front Endocrinol (Lausanne).* (2025) 16:1628612. 10.3389/fendo.2025.1628612 40771271 PMC12325020

[72] Wright VJ Schwartzman JD Itinoche R Wittstein J. The musculoskeletal syndrome of menopause. *Climacteric*. (2024) 27:466–72. 10.1080/13697137.2024.2380363 39077777

[73] Lu L Tian L. Postmenopausal osteoporosis coexisting with sarcopenia: the role and mechanisms of estrogen. *J Endocrinol*. (2023) 259:e230116. 10.1530/JOE-23-0116 37523234

[74] Curry ZA Beling A Borg-Stein J. Knee osteoarthritis in midlife women: unique considerations and comprehensive management. *Menopause* (New York, N.Y.). (2022) 29:748–55. 10.1097/GME.0000000000001966 35674654

[75] Davis SR Pinkerton J Santoro N Simoncini T. Menopause-biology, consequences, supportive care, and therapeutic options. *Cell.* (2023) 186:4038–58. 10.1016/j.cell.2023.08.016 37678251

[76] Magliano M. Menopausal arthralgia: fact or fiction. *Maturitas*. (2010) 67:29–33. 10.1016/j.maturitas.2010.04.009 20537472

[77] Klemm P Schulz N Boettger P Lange U. Heat therapy in rheumatic and musculoskeletal diseases - an overview of clinical and molecular effects. *Int J Hyperthermia.* (2024) 41:2322667. 10.1080/02656736.2024.2322667 38439192

[78] Wluka AE Cicuttini FM Spector TD. Menopause, oestrogens and arthritis. *Maturitas*. (2000) 35:183–99. 10.1016/s0378-5122(00)00118-3 10936736

[79] Strand NH D’Souza RS Gomez DA Whitney MA Attanti S Anderson MA et al. Pain during menopause. *Maturitas*. (2025) 191:108135. 10.1016/j.maturitas.2024.108135 39500125

